# Mass spectrometry locates local and allosteric conformational changes that occur on cofactor binding

**DOI:** 10.1038/ncomms12163

**Published:** 2016-07-15

**Authors:** Rebecca Beveridge, Lukasz G. Migas, Karl A. P. Payne, Nigel S. Scrutton, David Leys, Perdita E. Barran

**Affiliations:** 1 Michael Barber Centre for Collaborative Mass Spectrometry, School of Chemistry, Centre for Synthetic Biology of Fine and Speciality Chemicals, Manchester Institute of Biotechnology, University of Manchester, 131 Princess Street, Manchester M1 7DN, UK

## Abstract

Fdc1 is a decarboxylase enzyme that requires the novel prenylated FMN cofactor for activity. Here, we use it as an exemplar system to show how native top-down and bottom-up mass spectrometry can measure the structural effect of cofactor binding by a protein. For Fdc1^Ubix^, the cofactor confers structural stability to the enzyme. IM–MS shows the *holo* protein to exist in four closely related conformational families, the populations of which differ in the *apo* form; the two smaller families are more populated in the presence of the cofactor and depopulated in its absence. These findings, supported by MD simulations, indicate a more open structure for the *apo* form. HDX-MS reveals that while the dominant structural changes occur proximal to the cofactor-binding site, rearrangements on cofactor binding are evident throughout the protein, predominantly attributable to allosteric conformational tightening, consistent with IM–MS data.

## Abstract (web summary)

The decarboxylate enzyme Fdc1 requires a prenylated flavin mononucleotide co-factor for activity. Here, the authors use a variety of mass spectrometric techniques to observe the structural changes in this protein in response to co-factor binding.

## Graphical abstract

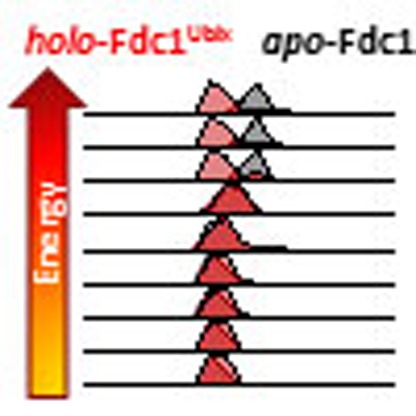

Decarboxylation reactions are common in nature, despite the fact they are difficult to achieve under ambient conditions. The reaction is made possible by decarboxylase enzymes, often making use of cofactors, including either organic molecules such as flavins, pyridoxal phosphate or thiamine pyrophosphate, and/or metal ions, for example, Mg^2+^, Fe^2+^ or Mn^2+^ (ref. 1). The recently discovered prenylated flavin cofactor that features in the Pad1/Fdc1 and UbiX/UbiD decarboxylase systems represents a new addition to this list^2^,^3^.

It has been previously demonstrated that both the *fdc1* and *pad1* genes are essential for the decarboxylation of phenylacrylic acids by spoilage yeasts and moulds such as *Saccharomyces cerevisiae* and *Aspergillus niger*; however, the precise role of each gene had remained enigmatic^4^,^5^. Recently, we have shown that Fdc1 is in fact the enzyme responsible for decarboxylation, although an active form of the recombinant protein can only be obtained by co-expression with either *pad1* or the corresponding *Escherichia coli* homologue *ubiX*^2^. The co-expressed Fdc1 protein (denoted Fdc1^UbiX^) possesses distinct features in the ultraviolet–visible spectrum that are absent in single-expressed Fdc1. Determination of the crystal structure of the *A. niger* Fdc1^UbiX^ revealed the presence of a modified flavin mononucleotide (FMN) cofactor bound to the protein in complex with Mn^2+^ and K^+^. This modified cofactor (prenylated FMN or prFMN) results from addition of a prenyl group to the N5–C6 atoms of FMN to form a fourth, non-aromatic ring. It is proposed that the prFMN cofactor supports decarboxylation of substrate by dipolar 1,3 cycloaddition, given the azomethine ylide character. Subsequent studies on UbiX/Pad have confirmed that these proteins are responsible for prFMN synthesis^3^. While Fdc1^UbiX^ could be readily crystallized, no crystals were attainable from recombinant Fdc1 produced in the absence of *ubiX* overexpression. It is hypothesized that this is due to an increase in conformational freedom of the *apo* protein, increasing the sampled conformational heterogeneity and decreasing the likelihood of crystallization. In this work, we present differences in the conformational dynamics of Fdc1 on cofactor binding. In the absence of any crystallographic reference structure for the *apo* form, we use complementary mass spectrometry (MS)-based approaches to determine the effect of prFMN binding; global conformational change is assessed with ion mobility (IM)–MS, whereas hydrogen–deuterium exchange-MS (HDX-MS) allows the changes to be localized to regions of the Fdc1 dimer interface. Molecular dynamics (MD) simulations were also carried out and together these results indicate that the cofactor confers stability to the enzyme.

With the use of nano-electrospray ionization (nESI)^6^, protein complexes can retain their native topology and stoichiometry on transfer into the gas phase^7^, an approach termed ‘native MS'^8^. Following desolvation from aqueous solution, the ensuing charge-state distribution provides mass and stoichiometric information, and can be used to infer some conformational preference for the protein or complex^9^. Native MS is highly appropriate to examine dynamic properties of proteins: it has no apparent bias towards a folded structure^10^. IM–MS facilitates visualization of the shape distribution of a given protein or protein complex in a form known as a collision cross section distribution (CCSD), which provides direct information of the size and conformational variability of a given system^11^. With IM–MS, it is possible to separate multiple conformational states^12^, for example, to observe how individual conformers are affected by ligand binding^13^. This positions it as highly complementary to X-ray crystallography; it cannot provide atomistic detail, but it can report on structurally dynamic systems and heterogeneic stoichiometries all in a single experiment, which is not reliant on successful crystal formation.

HDX-MS is also complementary to IM–MS, since it can also probe protein dynamics, allowing comparison between conformational changes observed *in vacuo* to those *in vitro*. When coupled with enzymatic digestion of the protein post deuteration, it provides structural information at a more localized level. A recent study carried out by Alverdi *et al*.^14^ used a combination of native MS, IM–MS and HDX-MS to further characterize the conformational rearrangement of PKG (cyclic guanosine 3′,5′-monophosphate (cGMP)-dependent protein kinase) in response to cGMP-dependent activation. IM results suggested that cGMP binding to PKG promotes a more extended structure, and HDX experiments allowed the localization of conformational change to the different domains. Data obtained from a combination of these MS techniques allowed the authors to propose a new structural model for the cGMP-induced activation of PKG.

In this work, we utilize three MS techniques, namely, native MS, IM–MS and HDX-MS, to characterize the effects of the prFMN cofactor, synthesized by the co-expressed enzyme (UbiX), on the structure of the decarboxylase Fdc1. Our experimental work is supported by MD simulations that examine the evolution of the structure of Fdc1^Ubix^ in the absence of solution and consider the corresponding change in ^DT^CCS_He_. Both the experiment and computation provide insights into the entire conformational ensemble of this enzyme, both in its active and inactive forms. This could not be achieved with standard structural biology approaches, since the *apo* form did not crystallize. The study emphasizes how MS can play a unique role in dynamic structural science by identifying how prFMN imprints new conformational properties into the Fdc1 protein. Both the approach and observations are of general significance within the context of the dynamic structure-defines-function paradigm, and in particular highlight the capability of a MS approach in establishing the role of cofactors on stabilising protein structure.

## Results

### Native mass spectrometry of Fdc1 and of Fdc1^Ubix^

**Figure 1:**
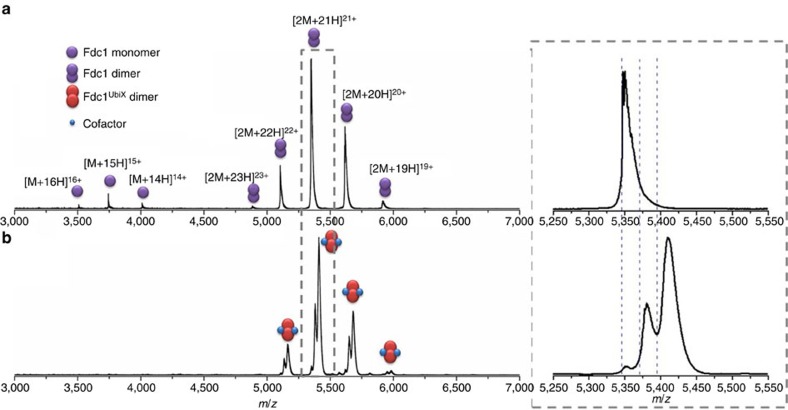
Mass spectrum of Fdc1 and Fdc1^Ubix^ displaying stabilising effects of the prFMN cofactor. (**a**) Native MS of 5 μM Fdc1 and (**b**) 10 μM Fdc1^UbiX^ showing some dissociation of the Fdc1 dimer into monomer, while all of the Fdc1^UbiX^ is present as a dimer. This figure has been adapted from Fig. 5b in the extended data set of Payne *et al*.^2^ here, reporting data over a wider *m*/*z* range. Both samples were sprayed from 100 mM ammonium acetate, pH 6.8. Right hand spectrum; an enlarged view of [2M+21H]^21+^. The measured mass of dimeric Fdc1 is 112,266.4±1.5 Da (expected 112,270 Da). The predicted masses are shown by blue dashed lines.

Native mass spectra are shown in Fig. 1, of Fdc1 Fig. 1a and Fdc1^UbiX^ Fig. 1b, sprayed from physiological-like conditions (100 mM ammonium acetate, pH 6.8). These data were used to support our previous study^2^. Fdc1, present only in the *apo* form, presents in charge states [2M+19H]^19+^ to [2M+23H]^23+^, with [2M+21H]^21+^ being the most intense. The native mass spectrum of Fdc1^UbiX^ shows a very slight narrowing in the charge-state distribution; although the most abundant ion is still [2M+21H]^21+^, the highest-charge state is now [2M+22H]^22+^. The first evidence in the study for the stabilization of the dimer by the cofactor is demonstrated by the monomer peaks in the Fdc1 spectrum, whereas all of the Fdc1^UbiX^ presents as a dimer. Whether these dissociated subunits reflect the solution-phase properties or are due to some dissociation of the complex in the mass spectrometer, the presence of the monomer in only the Fdc1 spectrum strongly indicates that this dimer is less stable than that of the Fdc1^UbiX^.

The Fdc1^UbiX^ spectrum in Fig. 1b, predominantly shows a species with two non-covalently bound cofactors. Approximately one-third of the ion intensity is attributed to the protein with one non-covalently bound cofactor and just a small amount of protein is observed without any bound cofactor. This heterogeneity in the protein complex may be due to incomplete cofactor occupancy in solution or to loss of the cofactor on desolvation (*vide infra*).

The measured mass of the Fdc1 dimer expressed in the absence of *ubiX* (112,266.4±1.5 Da) is in close agreement to the predicted mass of 112,270 Da (Fig. 1a). The replete Fdc1^UbiX^ bound to two prFMN molecules, which is dominant in the native mass spectrum, (Fig. 1b) has a measured mass of 113,609±45.3 Da, 294 Da larger than expected. Within the 4ZA4 structure, each monomer binds one manganese and two potassium ions, as well as many waters, and we can therefore attribute this extra mass to these six specified metal adducts, as well as one or two retained water/buffer molecules. Again, the crystallographic data that attributed electron density to a manganese atom in each active site was supported by electron paramagnetic resonance (EPR) experiments, where a characteristic Mn^2+^ signal was observed in the EPR spectra of Fdc1^UbiX^ (ref. 2). The measured mass for the co-expressed protein Fdc1^UbiX^ with no prFMN bound is 112,369±87 Da, slightly higher than for singly expressed Fdc1. Here, the increase in mass of ∼103 Da would corresponds well to two manganese adducts. When one cofactor is bound the difference between the expected (112,870 Da) and measured mass is higher again: ∼122 Da above what is predicted when the cofactor is in complex with the protein. This could be attributed to the retention of three potassium ions or, two waters and two potassium ions, as well as the two manganese ions, suggesting that these are more strongly bound than prFMN, and indicating that the prFMN depleted forms seen in MS indicate that some of this molecule may be lost during the ESI process, a point that we return to later.

Importantly, for all species, the predicted mass (with no retention of salt) corresponds to the left hand side of the peak (blue dashed lines, Fig. 1), while the apex of the peak corresponds to the adducted forms discussed above. These spectra show clearly that the Fdc1 dimer carries no cofactors and retains very little salt, whereas Fdc1^UbiX^ dimers carry either one or two cofactors of 525 Da along with a variety of unresolved adducts. An increase in salt retention occurs with bound cofactors, indicating that the addition of the cofactor favors the retention of counter ions, some of which are evidenced in the crystal structure.

### IM–MS reveals smaller conformers for Fdc1^Ubix^ than for Fdc1

**Figure 2:**
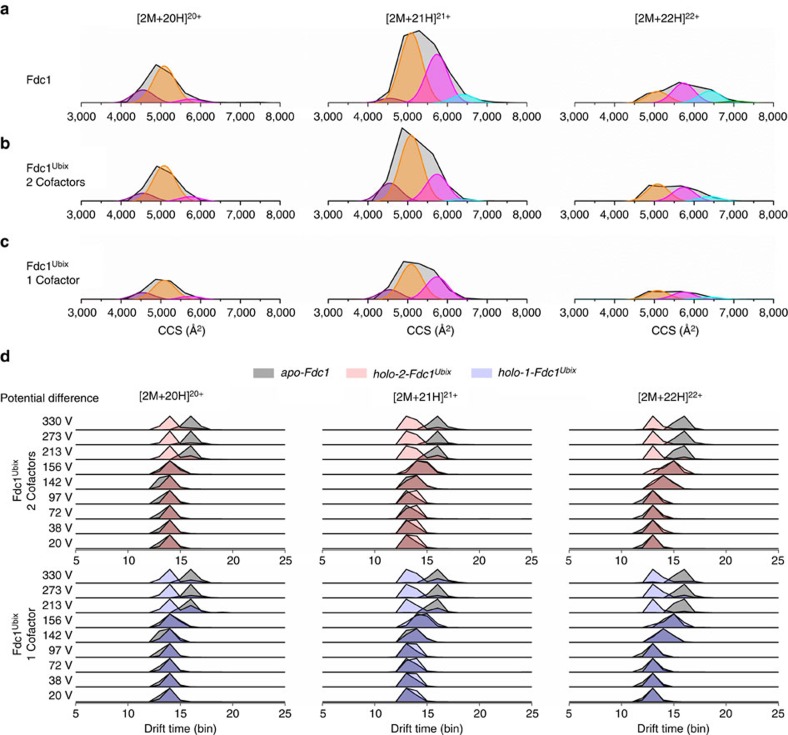
Collision cross section distributions of Fdc1 and Fdc1^Ubix^. (**a**) Arrival time distributions of the three main charge states of Fdc1, (**b**) Fdc1^Ubix^ with two bound cofactors and (**c**) with one bound cofactor. The purple curve corresponds to the conformation at 4,550 Å^2^, orange is 5,085 Å^2^, pink is 5,736 Å^2^ and cyan is 6,400 Å^2^. A green curve in the ATD of the *apo* form of [2M+22H]^22+^ corresponds to an additional conformational family centred on 7,042 Å^2^, which is eliminated by the presence of the cofactor. (**d**) ATDs of Fdc1 and Fdc1^Ubix^ at increasing cone voltages with two bound cofactors and one found cofactor. The peaks shaded grey represent the *apo*-Fdc1 protein and are overlaid onto each of the forms of Fdc1^Ubix^ protein: blue are *holo-1*-Fdc1^Ubix^ and red are *holo-2*-Fdc1^Ubix^.

^DT^CCS_He_ distributions of the three main charge states of Fdc1 are shown in Fig. 2: Fdc1 (Fig. 2a) and the cofactor-bound forms of Fdc1^UbiX^ (Fig. 2b,c) and in Supplementary Fig. 1. The cross sections of the [2M+20H]^20+^ ions range from ∼4,000–6,400 Å^2^ for Fdc1, or ∼4,000–6,000 Å^2^ for Fdc1^UbiX^, and the apex of the distribution is just under 5,000 Å^2^ for both forms of the protein; for this charge state, there are only small differences in the ^DT^CCS_He_ distributions of Fdc1 and different forms of Fdc1^UbiX^, and these indicate a compaction in the ligand-bound form, the higher ^DT^CCS_He_ distributions are not populated. For [2M+21H]^21+^, the lower value for the ions is ∼4,200 Å^2^ for all species; however, there is a difference between the upper value of Fdc1 (7,250 Å^2^) and Fdc1^UbiX^ (6,750 Å^2^). The apex of the peak is also different; for Fdc1, it is ∼5,250 Å^2^, whereas for Fdc1^UbiX^ it is ∼4,800 Å^2^. The differences in ^DT^CCS_He_ distribution are most prominent for the [2M+22H]^22+^ ion: Fdc1^UbiX^ displays two conformers centred ∼4,900 and 5,600 Å^2^, both at roughly equal intensity; for Fdc1, however, the smaller conformer is greatly reduced in intensity in comparison to the extended state, indicating a destabilization of the compact conformation in the absence of the cofactor. These IM–MS experiments are in agreement with native MS data presented above, both indicating a more compact structure in the presence of the cofactor.

Gaussian curves were fitted to the most intense CCSDs for the *apo* form and retained across other charge states and cofactor stoichiometries to highlight the distinguishable conformations with reference to the *apo* form. This procedure has been used previously for protein complexes^15^ and monomeric proteins^16^,^17^. The smallest conformer, shown by the purple curve centred on 4,550 Å^2^, contributes to the [2M+20H]^20+^ and [2M+21H]^21+^ charge states; it contributes more to [2M+21H]^21+^ Fdc1^UbiX^ than Fdc1, suggesting that this small conformation is stabilized in this charge state by the cofactor. The conformer centred on 5,085 Å^2^ (orange) is dominant in all forms of the [2M+20H]^20+^ and [2M+21H]^21+^ charge states. This conformer remains dominant in the [2M+22H]^22+^ charge state of Fdc1^UbiX^, but is of lower relative intensity in the same charge state of Fdc1, indicating that the cofactor stabilizes this ‘orange' conformation in [2M+22H]^22+^. A conformer centred on 5,736 Å^2^ (pink) makes a minor contribution to all forms of [2M+20H]^20+^, while to [2M+21H]^21+^ and [2M+22H]^22+^ it is of higher relative intensity in Fdc1 than Fdc1^UbiX^. The conformer centred on 6,400 Å^2^ (cyan) is also more intense in the higher-charge states of Fdc1 than Fdc1^UbiX^. An additional conformer centred on 7,042 Å^2^ of the [2M+22H]^22+^ ion is eliminated in the presence of the cofactor. So while the [2M+20H]^20+^ ion is similar for Fdc1 and Fdc1^UbiX^, in the higher-charge states the ‘orange' conformation is stabilized by the cofactor, and in the *apo* form it is replaced by the ‘pink' and ‘cyan' conformations, and an additional ‘green' conformation.

### MD shows the evolution of Fdc1^Ubix^ *in vacuo*

**Figure 3:**
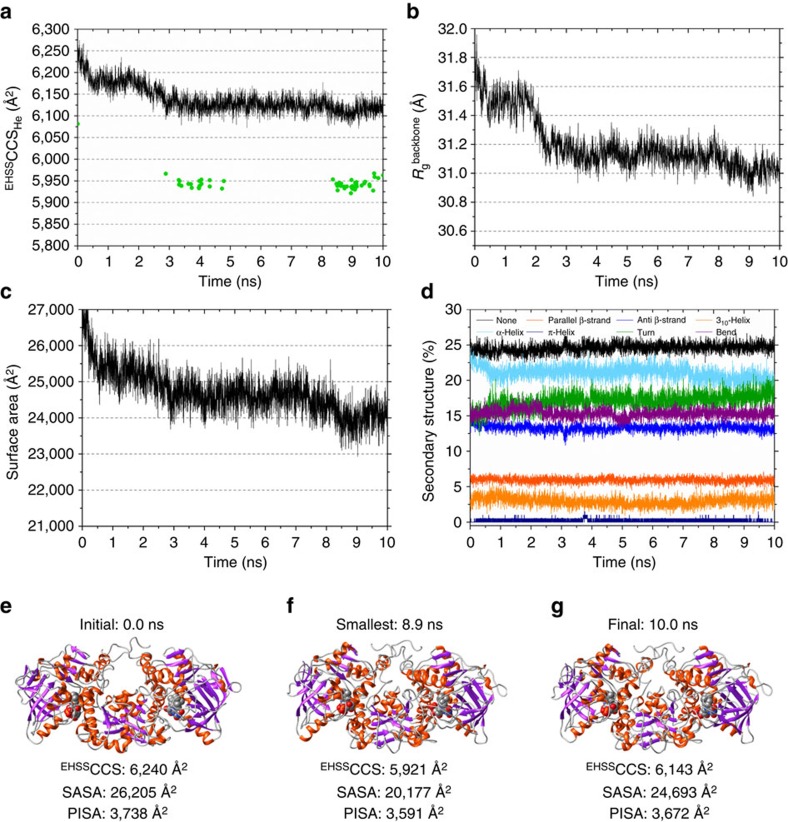
Molecular dynamics trends for the holo-Fdc1 at 350 K simulation. (**a**) Collision cross section (calculated using the ‘Exact Hard Sphere' method) versus time. The ^EHSS^CCS_He_ for minimized structures is depicted using the green dots; (**b**) backbone radius of gyration versus time; (**c**) solvent accessible surface area (SASA) versus time; and (**d**) secondary structure content versus time. Snapshots of: (**e**) the initial structure at 0 ns; (**f**) the smallest found structure by ^EHSS^CCS_He_ ; and (**g**) the final structure in the trajectory. The protein structure coloured by the secondary structure features: α-helix (orange), β-strand (purple) and coil (grey). Protein images made using UCSF Chimera^33^. Data for *holo*-Fdc1^UbiX^ at 300 K is available in the Supplementary Fig. 2.

To further explore the conformational dynamics of Fdc1^UbiX^, we performed *in vacuo* MD on the Fdc1^UbiX^ protein in the gas phase, starting from the crystal structure coordinates (Fig. 3). Results of the MD simulation are shown in terms of (Fig. 3a) the calculated exact hard sphere collision cross section (^EHSS^CCS_He_), (Fig. 3b) the radius of gyration (*R*_g_), (Fig. 3c) the solvent accessible surface area (SASA) of the protein and (Fig. 3d) the composition of secondary structural elements. Figure 3 and Supplementary Fig. 2 show the MD data of cofactor-bound Fdc1^UbiX^ at 350 and 300 K, respectively. The studies were carried out at different temperatures to imitate different cone voltages (*vide infra*).

During the simulation of Fdc1^UbiX^ at 350 K (Fig. 3), all measured parameters signify a collapse in the gas-phase structure. Between *t*=0 and *t*=10 ns, the ^EHSS^CCS_He_ decreases from 6,240 to 6,143 Å^2^ and the *R*_g_ decreases from 31.7 to 31 Å. The surface area of the complex decreases from 27,000 to 24,000 Å^2^, and to 20,177 Å^2^ for the smallest minimized conformer, representing a decrease of 11 and 23%, respectively. The interfacial area between the two monomers as calculated by PISA (Protein Interfaces, Surfaces and Assemblies)^18^ decreased by 2% between *t*=0 and *t*=10 ns, and 4% for the smallest model structure indicating little structural rearrangement at the interface of the complex and retention of the Fdc1^Ubix^ structure. The total α-helical content decreases marginally from 26.2 to 23.6%, while the β-sheet content decreases from 21.2 to 20.3% (Fig. 3d). A selection of structures were minimized to optimize the position of all atoms, resulting in significant reduction of the ^EHSS^CCS_He_ values down to 5,920 Å^2^ (green dots, Fig. 3a). When MD simulations of Fdc1^UbiX^ were carried out at 300 K (Supplementary Fig. 2), the reduction in *R*_g_ is less pronounced than at 350 K, and the ^EHSS^CCS_He_ measurements of the minimized structures correspondingly larger, but again are notably smaller than the raw crystal structure coordinates. The lowest ^EHSS^CCS_He_ found in these short simulations is 5,983 and 5,921 Å^2^ for Fdc1^UbiX^ at 300 and 350 K, respectively. Figure 3e–g and Supplementary Fig. 2e–g depict three representative structures from each trajectory. The data here indicates a higher degree of collapse of the protein at 350 K than at 300 K.

Following 10 ns of MD and minimization, the ^EHSS^CCS_He_ of Fdc1^Ubix^ reduces by 6.1%, to a value that is close to the apex of the pink conformer observed experimentally, (Fig. 2). This is similar to previously published findings, where *in vacuo* MD of large proteins starting from crystal structure coordinates results in significant conformational tightening^11^,^19^. All IM spectrometry experiments last significantly longer (milliseconds) than the nanosecond time scales of these atomistic simulations, allowing much more time for gas-phase collapse to a tighter packed form. This closer packing is entirely expected due to the increased contribution from favourable electrostatic interactions in the absence of mitigating solvent^20^, and no competing effects from repulsive coulombic interactions for the large protein as it presents with such low *z*. We also calculated the CCS_He_ of the smallest conformation that the Fdc1 dimer could possibly adopt, by assuming that this conformation is spherical in shape with a density of *ρ=*0.904 Da Å^−3^ (ref. 9). This smallest theoretical cross section is 3,430 Å^2^, ∼14% smaller than the smallest ^DT^CCS_He_ measured in our mobility experiments. Our analysis shows how the conformations of Fdc1 in the gas-phase range from very compact to more extended, indicative of a flexible protein in solution, as well as the width of the CCSDs, which imply that the protein is also changing shape on the time scale of the mobility experiment. This is supported by our MD simulations that the protein to sample many conformations even in 10 ns.

### MS and IM-MS probe the influence of prFMN

A key question is why we observe a proportion of Fdc1^Ubix^ carrying less than two cofactors. The possible reasons are that (i) this reflects the cofactor occupancy in solution or (ii) some cofactor dissociates from the protein during desolvation. The crystal structures^2^ were modelled with 100% cofactor occupancy, but whether this reflects the solution-phase character, or is due to preferential crystallization of the dimer with two cofactors, remains unknown. Our IM–MS results show that the different forms of Fdc1^UbiX^ have similarly shaped ^DT^CCS_He_ distributions for the same charge states, regardless of the number of non-covalently bound cofactors. An explanation for this is that some cofactor is lost on desolvation, but the protein retains the conformation adopted when in complex with the cofactor, a templating effect we have reported previously^21^ due to the time scale for the conformational change in the protein complex being significantly longer than the experimental time scale of the IM–MS process. This is supported by the fact that in our analysis of Fdc1^UbiX^, we are always able to resolve a species at *m*/*z* 525 that corresponds exactly to the mass of the cofactor even under the most gentle desolvation conditions. Further evidence that the cofactor is lost during desolvation is found when on modulating the harshness of the ionization process, we can alter the proportion of single- and double-cofactor-bound protein (Supplementary Fig. 3). By increasing the voltage applied to the sample cone, we cause the protein to experience a larger potential difference during desolvation, which results in harsher ionization conditions. When the cone is increased to 200 V, the ratio of the intensities of singly bound to doubly bound is ∼0.75, up from ∼0.66 at a cone voltage of 60 V. This native IM–MS data indicates that the *holo* form is dominant and more rigid than the *apo* form, but also that prFMN is somewhat labile and can dissociate from the protein. Under similar nESI–MS conditions, a haem cofactor has been shown to be fully retained by myoglobin and haemoglobin^22^, and FMN similarly is reported to be fully retained in glutamate synthase^23^ and flavodoxin^24^.

Increasing the energy of the ions in the desolvation stage of the instrument can cause proteins to deform, as well as induce fragmentation as alluded to above, and we performed such experiments on Fdc1^Ubix^ and Fdc1. The effect of such collisional activation on the gas-phase conformation of the intact complexes is shown in Fig. 2d. As the potential between the cone and the next sampling orifice is increased, Fdc1 adopts more elongated conformations between 156 and 213 V of source potential whereas Fdc1^Ubix^ maintains a more constant drift time up to the highest potentials applied, strongly indicative of a more stable conformation. Interestingly, the single cofactor and cofactor deplete forms of Fdc1^Ubix^ also retain a compact form to the most elevated voltages, again suggesting that prFMN is lost at this stage and that the stabilized *holo* form of the protein remains, this is also consistent with the retention of metal ions even in the prFMN deplete forms as discussed above. MD shows the behaviour of the cofactor present Fdc1^Ubix^ is highly similar to simulations performed, where we removed the cofactor (Fig. 2; Supplementary Fig. 2; and data not shown).

### HDX-MS locates conformational change due to prFMN

**Figure 4:**
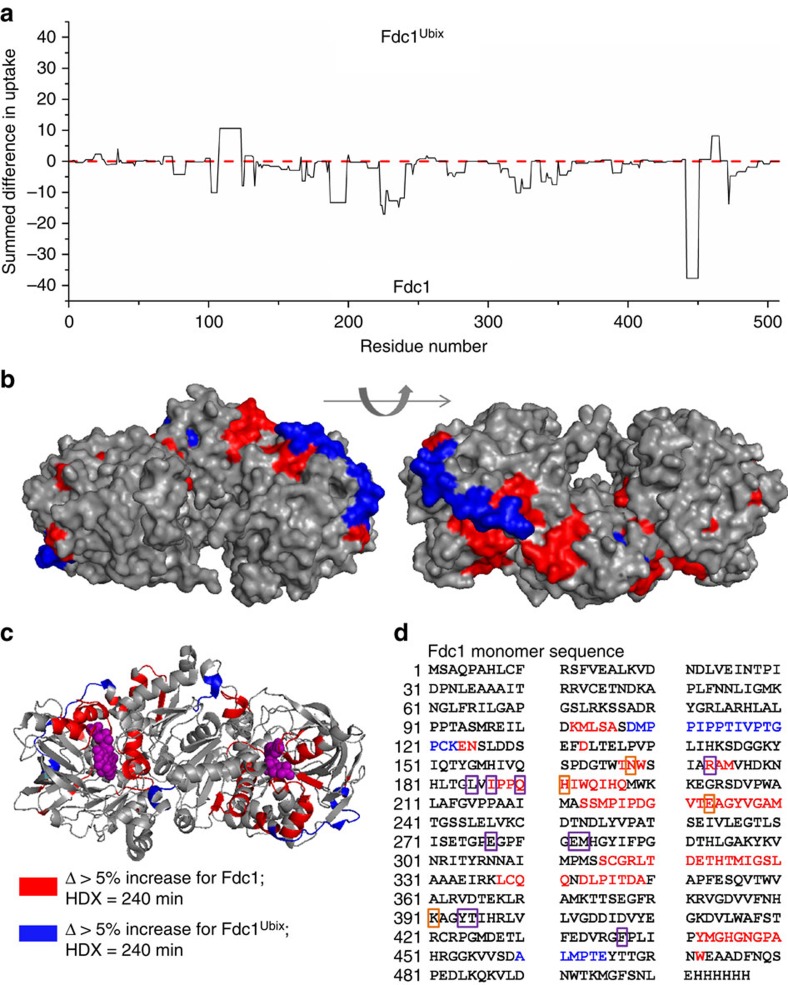
Summary of results from HDX-MS experiments. (**a**) Summed difference in deuterium uptake between Fdc1^Ubix^ (top) and Fdc1 (bottom) from the N to the C terminus of a single Fdc1 unit. (**b**,**c**) The deuterium uptake mapped onto the structure of Fdc1^Ubix^ (PDB ID: 4ZA4). (**b**) depicts two surface representations transformed through 180°, whereas (**c**) shows a single cartoon representation of secondary structure elements. Differences in deuterium uptake are coloured according to the key with the cofactor prFMN shown in purple. (**d**) shows the same exchange data mapped directly to the linear sequence, the residues that interact with the cofactor are outlined in purple, while the residues that are involved in Mn^2+^ binding are outlined in orange.

The difference in deuterium uptake between Fdc1 and Fdc1^UbiX^ summed over all time points is reported in Fig. 4a, and the relative fractional uptake of deuterium at discrete time points for Fdc1^UbiX^ (top) and Fdc1 (bottom) in Supplementary Fig. 4. The coverage map and several uptake plots of selected peptides are shown in Supplementary Figs 5 and 6, respectively, for each form of the protein 95% coverage was obtained. Across the majority of the peptides sampled there is an overall increase in mass when comparing the uptake of Fdc1 against Fdc1^UbiX^, indicating a more dynamic structure for Fdc1 in agreement with both MS and IM–MS experiments. In particular, there is a higher uptake for Fdc1 in the regions containing residues 187–197, 223–240 and 441–450. X-ray crystallography has shown that H191 is involved in the binding of Mn^2+^ and Q190 is involved in the binding of the cofactor, which correlates with the HDX data.

The fact that the cofactor is not present and that we cannot observe Mn^2+^ binding in *apo*-Fdc1 is a possible explanation for this apparent destablization/increased solvent exposure of the *apo* protein around residues 187–197. E233 is also involved in Mn^2+^ binding that could account for the increased dynamics of residues 223–240. Other residues involved in Mn^2+^ binding are N168 and K391, both of which are within regions of increased uptake for Fdc1 compared with Fdc1^UbiX^, albeit at a lower level. E282, E277 and R173 are other residues known to be associated with the bound cofactor and these are also within regions of higher HDX in the absence of the cofactor. Interestingly, there are some regions within Fdc1^UbiX^ that have an increase in deuterium uptake in comparison with Fdc1: residues 108–123 and 460–465. The latter areas are in close proximity to regions that have an increased deuterium uptake for Fdc1, suggesting that in the absence of prFMN, residues around the cofactor-binding site are exposed to the solvent, and a conformational switch occurs driving the regions containing residues 108–123 and 460–465 into the centre of the protein.

Differences in deuterium uptake are visualized by plotting regions of changed mass at the peptide level onto the crystal structure of Fdc1^UbiX^ (PDB ID: 4ZA4), with the surface elements displayed in Fig. 4b and Supplementary Fig. 7, and the secondary structure elements shown in Fig. 4c. Significant variations in behaviour are found in close proximity to the cofactor-binding site (highlighted in Fig. 4d). These tend to be at the end of α-helices, in regions that are leading to disordered loops, indicating a change in stability of secondary structure elements on binding of the cofactor. This can also be seen in Supplementary Fig. 8. For example, the predominantly hydrophobic patch over residues 227–238 has an uptake of >5%, wherein 227–232 consists of a disordered loop and 233–238 are part of an α-helix that extends to the glycine (242). It is feasible that in the *apo* form the disordered loop extends at least to the glycine at residue 238 and the helix is correspondingly shorter. Another region of destablization corresponds to the N-terminal helix of Fdc1^UbiX^ spanning residues 5–20. There is an uptake in deuterium of >5% from residues 7–16, suggesting a reduction of secondary structure for this N-terminal region. It is important to consider that while the most appropriate way to display the difference in deuterium uptake is to plot it onto the Fdc1^UbiX^ structure, this will not correspond exactly to that of *apo*-Fdc1, and indeed our results show this. It is likely that *apo*-Fdc1 has reduced overall secondary structure content compared with the *holo* form, due to the location of the uptake differences at the ends of α-helices, suggesting that these are more dynamic and less configured in the absence of prFMN.

Uptake increases at short time points for Fdc1 (pink) and Fdc1^UbiX^ (cyan) are in close proximity to one another, either in the linear sequence or the position of the areas (Supplementary Fig. 7). Visualization of the surface of the crystal structure (Fig. 4b; Supplementary Fig. 7) illustrates differences in uptake both close to, and distant from, the cofactor-binding region. This indicates not only a conformational change in the areas that bind the cofactor, but also an allosteric change in conformation in areas distant from the cofactor. This insight to the effect of prFMN binding is possible, since HDX-MS is an approach that allows equal visualization of the *apo* and *holo* states of this protein, and how they behave as a function of time, in contrast to the snapshot of only the *holo* state found crystallographically.

### Conclusions and outlook

Here we have used three MS-based techniques to delineate how the structure and dynamics of Fdc1 are affected by the binding of prenylated FMN. Native MS results confirm the stoichiometry of the protein complex; when Fdc1 is expressed alone (that is, the absence of *UbiX* overexpression) it is detected only in the *apo* form, and presents mainly as a dimer with a charge state range (Δ*z*) of 5, with some low-intensity monomer peaks. When Fdc1 is co-expressed with *UbiX* the monomer peaks are eliminated, indicating a more stable structure in the presence of the ligand, since the dimer interaction is stronger. IM–MS detects four closely related conformational families centred on ^DT^CCS_He_ values of 4,550, 5,085, 5,736 and 6,400 Å^2^ that are populated differentially depending on the presence of the cofactor; the smallest two families are more dominant in the presence of the cofactor, indicating stabilization of the compact form of the quaternary fold. The two more extended families are of higher intensity in *apo*-Fdc1 than Fdc1^UbiX^, demonstrating the more dynamic nature of the protein in the absence of the cofactor. Further, an additional conformational family is observed in the CCSD of the *apo*-Fdc1 [2M+22H]^22+^ ion at 7,400 Å^2^ that is eliminated in the presence of the cofactor. The disappearance of peaks corresponding to the monomeric species in Fdc1^UbiX^ as shown by native MS and the smaller CCS measurements of Fdc1^UbiX^ *c.f. apo*-Fdc1 in IM–MS experiments demonstrate that the cofactor confers stability to the dimer and promotes more compact conformations. With HDX, we have (i) observed overall conformational tightening in the presence of the cofactor in solution and (ii) been able to localize regions of the protein where the alterations in dynamics occur. There are changes in deuterium uptake close to the cofactor binding and Mn^2+^-binding sites, suggesting conformational differences in these regions. The protein also appears to undergo allosteric changes in the presence of the cofactor, shown by differential deuterium uptake in regions distant from the cofactor-binding site. There is very little change in deuterium uptake on the dimer interface between the *apo* and *holo* forms of the protein, suggesting that the monomer species observed via MS are not transferred from solution, and likely arise from dissociation during the ionization process. Previous to the research presented in this paper, it was unknown how the residues moved in response to the cofactor binding. HDX-MS experiments, however, provide information on the change in solvent accessibility of localized regions of the protein.

Overall, we have observed differences in the *apo* and *holo* forms of the Fdc1 protein; specifically, the protein is more dynamic in the *apo* form and has less rigid tertiary and quaternary structure. This could not be observed via X-ray crystallography, since no crystals could be obtained of *apo*-Fdc1. This lack of crystallization may be due to the increased flexibility that we have observed in our experiments. Here we have a comparison between *apo*- and *holo*-Fdc1 that is equally weighted for both forms of the protein; the data do not preferentially report on the cofactor-bound form, as for other methods. We demonstrate the complementarity of MS to crystallographic approaches and in particular this study emphasizes its benefits in its use to study dynamic protein complexes and the effect of cofactors on stabilising protein fold.

## Methods

### Expression and purification of Fdc1 and Fdc1^UbiX^

The codon optimized *A. niger* fdc1 gene (Genscript) was cloned into the NdeI and XhoI sites of pET30a, while *E. coli* ubiX was cloned into the NdeI and XhoI sites of pET21b. *A. niger* fdc1 pET30a was transformed into *E. coli* BL21(DE3) with or without ubiX pET21b. Cells were grown at 37 °C/180 r.p.m. in LB broth supplemented with 50 μg ml^−1^ kanamycin (single expressed) or with both 50 μg ml^−1^ kanamycin and 50 μg ml^−1^ ampicillin (co-expressed). Cells were induced with 0.25 mM IPTG and supplemented with 1 mM MnCl2 at mid-log phase and, grown overnight at 15 °C/180 r.p.m. Cells were collected by centrifugation (4 °C, 7,000*g* for 10 min) and the pellets resuspended in buffer A (200 mM NaCl, 1 mM MnCl2, 50 mM Tris pH 7.5) supplemented with lysozyme, RNase, DNase (Sigma) and complete EDTA-free protease inhibitor cocktail (Roche). Cells were lysed by French press at 20,000 psi and the lysate clarified by centrifugation at 125,000*g* for 90 min before being applied to a Ni-NTA agarose column (Qiagen). The column was washed with three column volumes of buffer A containing 10 mM imidazole and the protein eluted in 1 ml fractions with buffer A supplemented with 250 mM imidazole. Fractions containing pure protein were identified by SDS–polyacrylamide gel electrophoresis analysis, pooled and buffer exchanged using a 10-DG desalting column (Bio-Rad) equilibrated 100 mM NaCl, 1 mM MnCl2, 25 mM Tris pH 7.5 to remove the imidazole. The protein concentration of *A. niger* Fdc1 was estimated from its absorbance at 280 nm (recorded with a Cary UV–Vis spectrophotometer) using *ɛ*280=68,870 M^−1^ cm^−1^ (calculated from the primary amino-acid sequence using the ProtParam program on the ExPASy proteomics server). The protein was aliquoted and flash-frozen until required.

### MS experimental conditions

Ten-DG desalting columns (BioRad) were used to buffer exchange the proteins into 100 mM ammonium acetate. Native MS spectra were acquired on a Synapt G2 instrument (Waters, Manchester, UK) with a nESI source. Mass calibration was carried out by separately infusing NaI cluster ions. Solutions were ionized by applying a positive potential through a platinum wire (thickness 0.125 mm, Goodfellow) that was inserted into a thin-walled glass capillary (inner diameter 0.9 mm, outer diameter 1.2 mm, World Precision Instruments, Stevenage, UK) that was pulled to a nESI tip in house with a Flaming/Brown micropipette puller (Sutter Instrument Co., Novato, CA, USA). Fdc1 samples (5–10 μM) were sprayed from 100 mM ammonium acetate pH, 6.8. Capillary voltage 1.6 kV, sample cone voltage 80–90 V, extractor cone voltage 3 V, source temperature 50 °C, backing pressure 5 mbar, Trap gas flow 0.4–5 ml min^−1^. Data were processed using MassLynx V4.1 software (Waters, Manchester, UK) and Origin 9.0 (OriginLab Corporation, USA).

### Ion mobility–mass spectrometry

IM–MS is a gas-phase electrophoretic technique that can be coupled to a mass spectrometer. A typical IM experiment involves ions being trapped and then pulsed into a chamber known as a drift tube, across which is applied a weak electric field that draws the ions through the tube. The drift tube is filled with an inert gas, in this case helium, which collides with the ions, hindering their passage through the drift tube. Larger ions will experience more frequent collisions with the buffer gas and will hence be slowed down to a greater extent. By measuring the arrival time of the ion and the charge present on it via subsequent MS, the rotationally averaged collision cross section can be directly calculated.

The mobility of an ion (*K*_0_) is determined as the ratio of the drift velocity (*v*_d_) and applied electric field (*E*). The ^DT^CCS_He_ can then be calculated on the basis of equation (1);


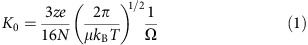


where *z* is the charge state of the ion, *e* is the elementary charge, *N* is the gas number density, *μ* is the reduced mass of the ion-neutral pair, *k*_B_ is the Boltzman constant, *T* is the gas temperature and *K*_0_ is the reduced mobility (the measured mobility *K* standardised for pressure and temperature to 273.15 K and 760 torr).

IM–MS experiments were carried out on a Waters Q-ToF I instrument that was previously modified in house to include a 5.1-cm drift tube that has been described elsewhere^10^. nESI was used to produce ions, with a capillary voltage of 1.6 kV and a source temperature of 80 °C. Tips were prepared as above. The pressure and temperature of helium in the drift cell were ∼4 torr and 30 °C, respectively. Measurements were recorded at six different drift voltages from 50 to 20 V. The precise pressure and temperature was recorded for every drift voltage and used in the calculations of ^DT^CCS_He_ values. Each experiment was performed in triplicate. Data were analysed using MassLynx v4.1 software, Origin 9.0 and Microsoft Excel. Ion arrival time distributions were recorded by synchronisation of the release of ions into the drift cell with mass spectral acquisition.

The ^DT^CCS_He_ distribution plots are derived from raw arrival time data using equation (2) below^25^.


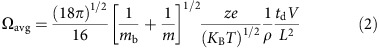


Where *m* and *m*_b_ are the masses of the ion and buffer gas, respectively; *z* is the ion charge state; *e* is the elementary charge; *K*_B_ is the Boltzmann constant; *T* is the gas temperature; *ρ* is the buffer gas density; *L* is the drift tube length; *V* is the voltage across the drift tube; and *t*_d_ is the drift time.

The raw arrival time output (*t*_a_) includes time the ions spend outside of the drift cell, but within the mass spectrometer, known as the dead time (*t*_0_). The value for *t*_0_ is calculated by taking an average value of the intercept from a linear plot of average arrival time versus pressure/temperature and was subtracted from the arrival time to calculate drift time (*t*_D_):


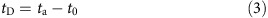


### Modelling of smallest possible CCS

This procedure has been described elsewhere^9^. In brief, the lower boundary was calculated by assuming that the globular form of the protein is approximately spherical in shape with a density of *ρ*=0.904 Da Å^−3^. The volume of the protein sphere can be calculated via *V*=*M*_w_/*ρ*, since the molecular weight *M*_w_ of the protein is also known. The radius of the sphere is therefore *r*=(3*V*/4*π*)^1/3^. The CCS of a sphere of this radius is given by equation 4:


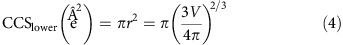


A scaling factor of 1.19 is then applied to this value for the conversion from geometric size to CCS in helium as outlined in ref. 26. These theoretical values are highly approximate and do not take into consideration proline residues, disulphide bridges, or other non-covalent interactions or restrictions. Instead, they serve as upper and lower boundaries with which experimental data can be compared.

### Homology modelling of Fdc1

The starting structure was obtained from RCSB Protein Data Bank (PDB ID: 4ZA4)^2^, missing residues were modelled using MODELLER based on the sequence alignment obtained from BLAST^27^. The resultant protein models were subsequently minimized and the most energetically favourable structure was selected for further computational simulations.

### Parameterization of prFMN ligand

Simulation parameters for the prFMN cofactor were derived by calculating atomic charges from multiple conformations using RED server according to the RESP model^28^. Ligand parameters were generated using antechamber module in Amber 14 (ref. 29).

### Molecular dynamics

MD simulations were performed using the sander module in Amber 14 for Fdc1^UbiX^. After adding hydrogen atoms, the protein was minimized *in vacuo* using an ‘infinite' radial cutoff of 999 Å and the amber14SB force field was used. The system was then gradually heated to 300 and 350 K using a weak coupling coefficient of 2 ps^−1^ with a time step of 2 fs. The SHAKE algorithm was used on all bonds involving hydrogen atoms. Following heating and equilibration, 10 ns of dynamics was carried out for each protein at 300 and 350 K; the higher temperature was chosen to imitate harsher ionization conditions the ion might experience at high cone voltage. The MD results were analysed using the cpptraj module. The conformational rearrangement of the dimer was monitored using the backbone *R*_g_, SASA, secondary structure (DSSP)^30^ and PISA area in CCP4 (refs 18, 31).

### Calculation of CCSs

Theoretical collision cross sections were calculated for each MD frame (every 2 ps) using the exact hard sphere model, as implemented in EHSSrot software^32^. A subset of structures was selected based on the *R*_g_, SASA and initial ^EHSS^CCS_He_ values; a total of 60–70 structures were selected for further energy minimization from each simulation run. The resulting minimized structures were then re-evaluated using the EHSS method to determine their corresponding ^EHSS^CCS_He_.

### HDX-MS experimental conditions

Fdc1 and Fdc1^UbiX^ solutions were prepared at 20 μM. Deuterium labelling and quenching were automatically performed using the CTC PAL sample manager (LEAP Technologies, Carrboro, NC, USA). The samples were first diluted 20-fold with 10 mM phosphate in 99.99% deuterium oxide, pH 6.6 (pD 7.0), and incubated for 0, 0.25, 5, 60 and 240 min at 20 °C. Quenching of the labelled samples was by the addition of an equal volume of pre-chilled 100mM phosphate pH 2.5. All labelling time points were analysed in triplicate. A measure of 50 μl of sample was injected on a nanoACQUITY UPLC system with HDX technology (Waters). Online pepsin digestion was performed for 1 min at 20 °C on a Waters Enzymate immobilized BEH pepsin column (2.1 × 30 mm). The peptides were separated on a UPLC BEH C18 column (Waters) at 0 °C. Peptides were separated with a 7-min linear acetonitrile gradient (8–35%) containing 0.1% formic acid at 40 μl min^−1^. Mass spectra were acquired on a SYNAPT G2-Si HDMS in MS^E^ mode with a *m*/*z* range of 50–2,000. Non-deuterated peptides were identified with ProteinLynx Global Server software 3.1 (Waters). DynamX 2.0 software (Waters) was used to filter the peptides and to generate deuterium uptake data. Origin 9.0 was used to create the plots.

### Data availability

The data and the essential metadata that support this study are available from the corresponding author on request.

## Additional information

**How to cite this article:** Beveridge, R. *et al*. Mass spectrometry locates local and allosteric conformational changes that occur on cofactor binding. *Nat. Commun.* 7:12163 doi: 10.1038/ncomms12163 (2016).

## Supplementary Material

Supplementary Information Supplementary Figures 1-8 and Supplementary Reference
