# Supplementary material for: Mass spectrometry locates local and allosteric conformational changes that occur on cofactor binding
Source: Nat Commun. 2016 Jul 15;7:12163. doi: 10.1038/ncomms12163 (PMC4947166; doi:10.1038/ncomms12163)
Supplement: Supplementary Information — Supplementary Figures 1-8 and Supplementary Reference [file ncomms12163-s1.pdf]

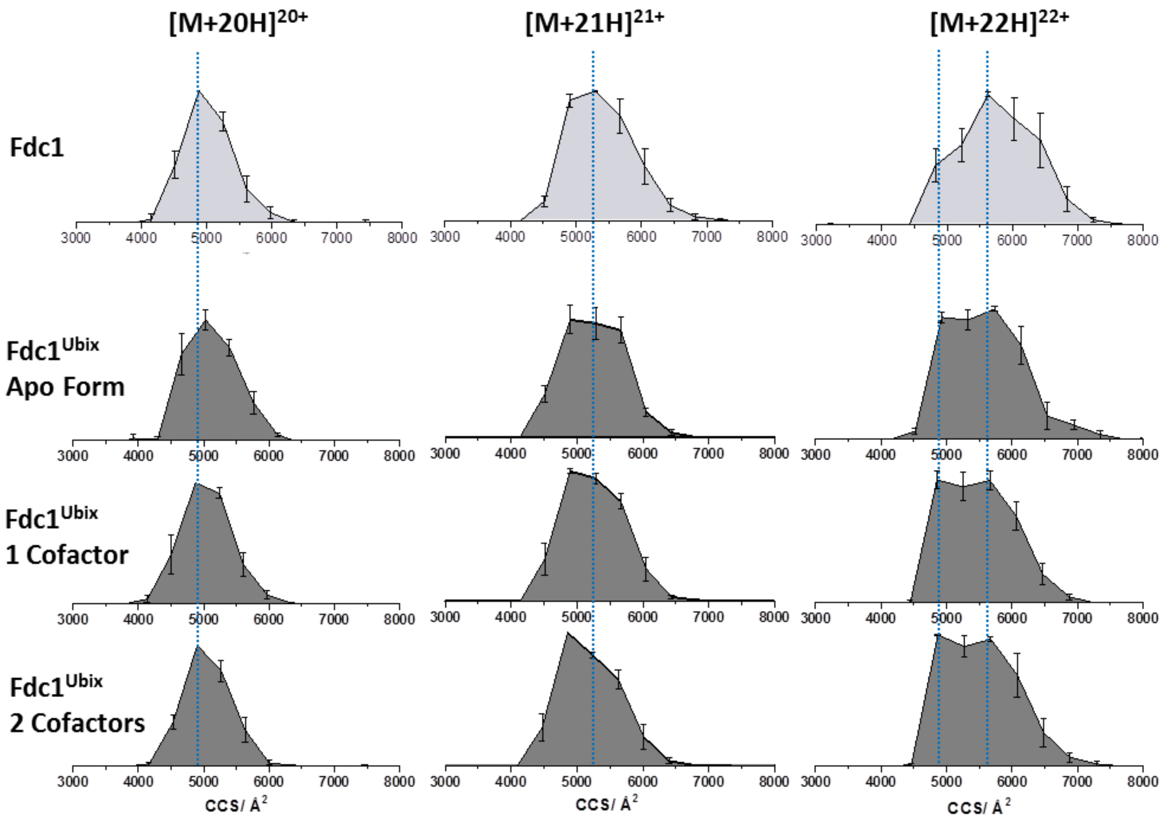

Supplementary Figure 1: CCS distributions of Fdc1 and Fdc1^{Ubix}. The distribution of CCS for the three main charge states of Fdc1 and also of Fdc1^{Ubix} containing none, one and two bound cofactors are shown.

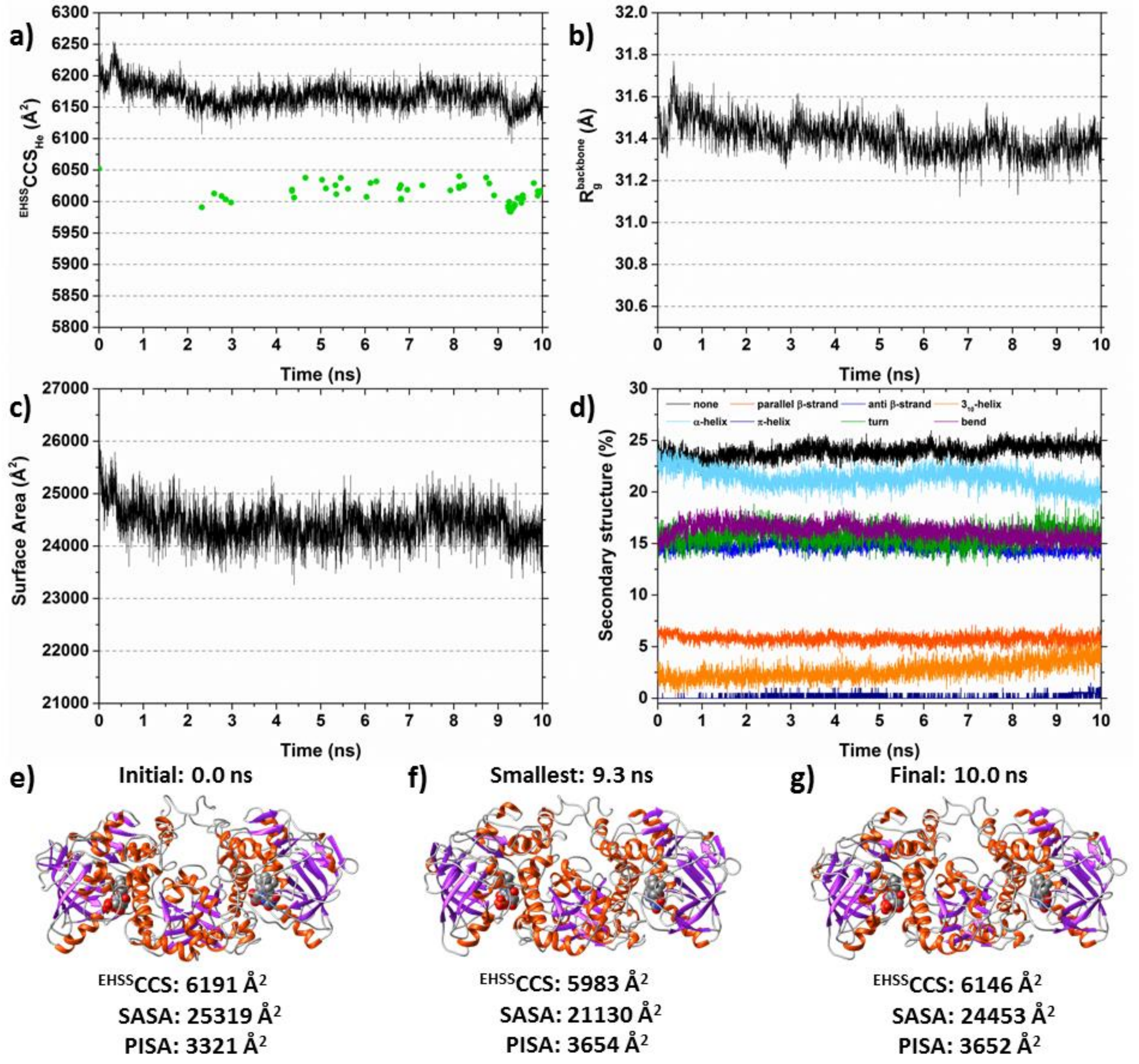

Supplementary Figure 2: Molecular Dynamics trends for the *holo*-Fdc1 at 300 K simulation. a) Collision Cross Section (calculated using the 'Exact Hard Sphere' method) *vs* time. The $^{EHSS}CCS_{He}$ for minimised structures is depicted using the green dots; b) backbone Radius of Gyration *vs* time; c) Solvent Accessible Surface Area (SASA) *vs* time; d) Secondary structure content *vs* time. Snapshots of the initial structure at 0 ns (e), the smallest found structure by $^{EHSS}CCS_{He}$ (f) and final structure (g). Protein structure coloured by the secondary structure features: α-helix (orange), β-strand (purple) and coil (gray). For further methodological details see main text, protein images made using UCSF Chimera [1].

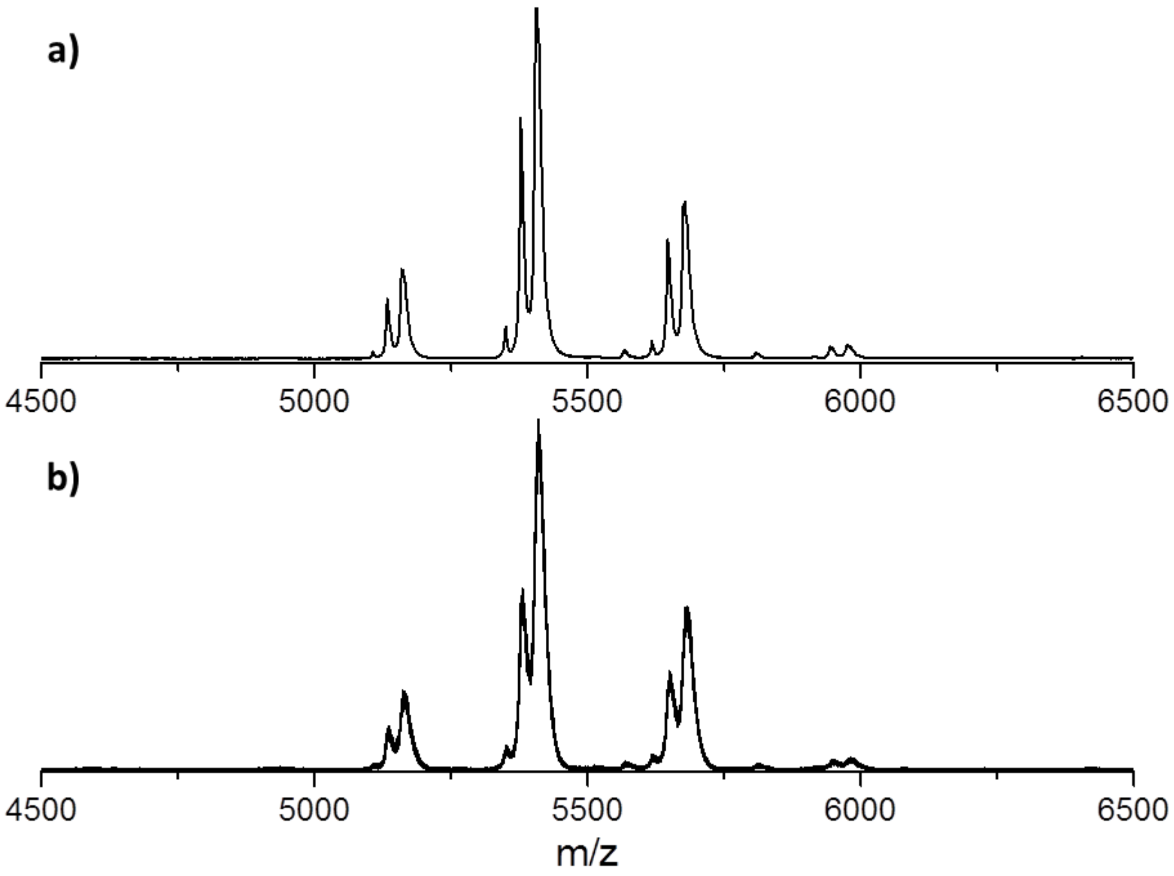

Supplementary Figure 3: Mass spectrum of Fdc1^{Ubix} at two source sampling cone voltages. a) Fdc1^{UbiX} with cone voltage = 200V (harsh), and b) cone voltage = 60 V (softer). The ratio of doubly bound to singly bound cofactor is altered by altering the cone voltage, the lower the voltage the more cofactor is retained. This data was acquired on a Synapt G2 HDMS mass spectrometer.

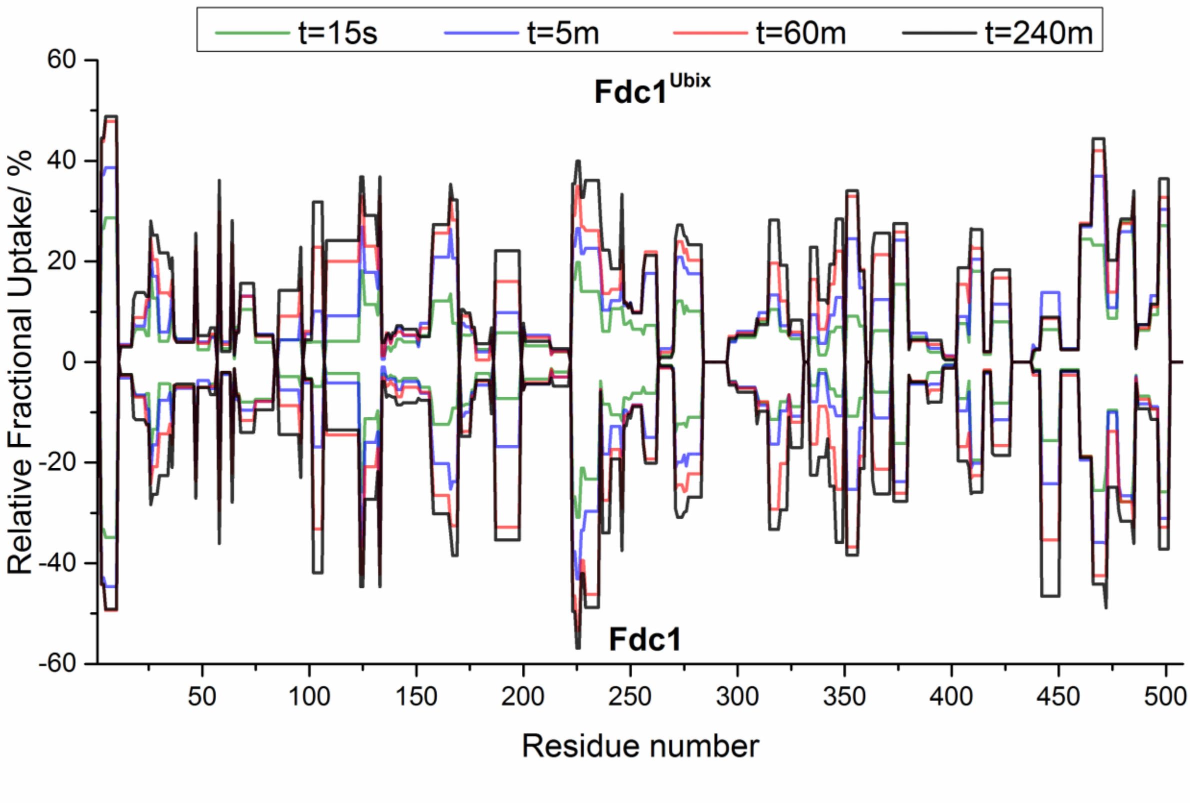

Supplementary Figure 4: Relative fractional uptake of deuterium for unique peptides along the polypeptide sequence of a single Fdc1 unit. Fdc1^{UbiX} (top) and Fdc1 (bottom) at t = 15s (green), t = 5m (blue), t = 60m (red) and t = 240m (black). Areas of the sequence for which no coverage could be achieved are denoted as uptake = 0.

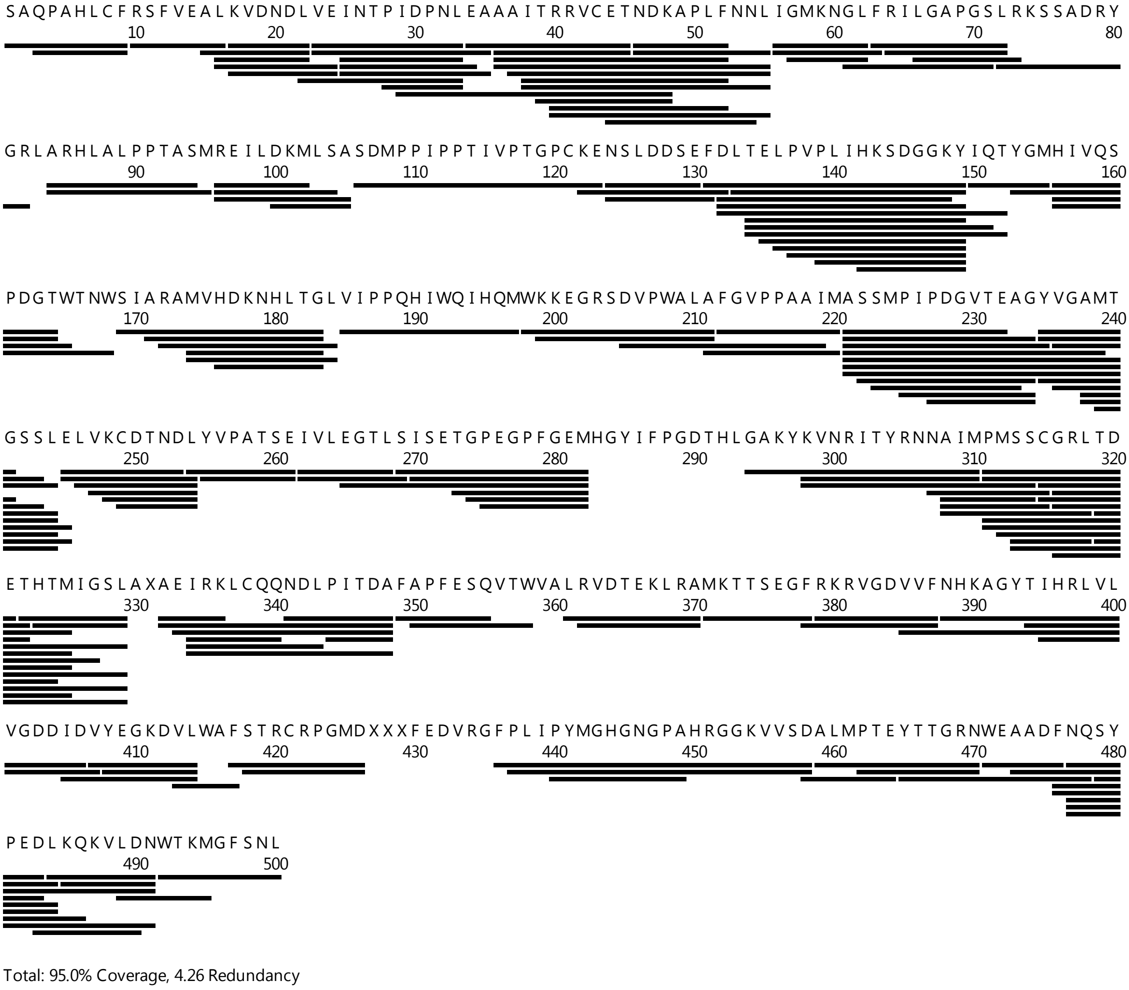

Supplementary Figure 5: Peptides which have been identified at each time point in the HDX exposure, providing the accessible sequence of Fdc1^{Ubix}. Total coverage 95% of the primary sequence.

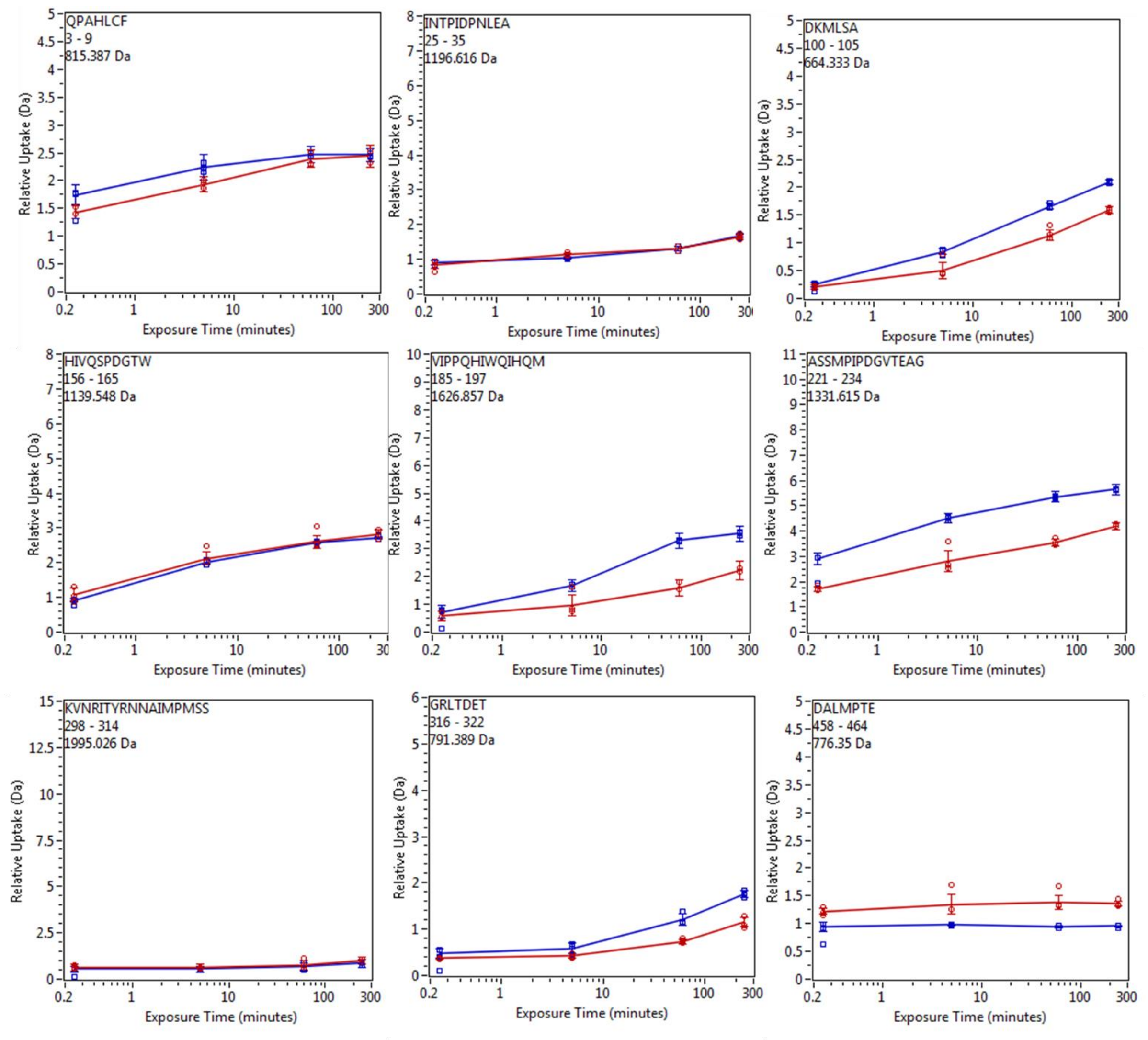

Supplementary Figure 6: Deuterium uptake plots of Fdc1 and Fdc1^{Ubix}. Deuterium uptake plots of selected peptides along the incubation time course for identical peptides from Fdc1 (blue) and Fdc1^{UbiX} (red).

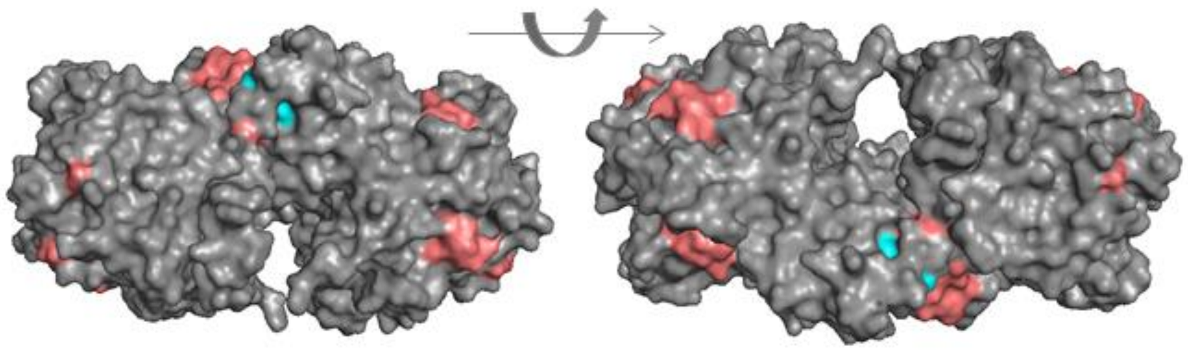

Supplementary Figure 7: The deuterium uptake mapped onto the structure of Fdc1^{Ubix} (PDB ID: 4ZA4) shown as a surface representation. Differences in deuterium uptake at t=0.25 minutes are coloured according to the following; Δ > 5% increase for Fdc1; pink. Δ > 5% increase for Fdc1^{UbiX}; cyan.

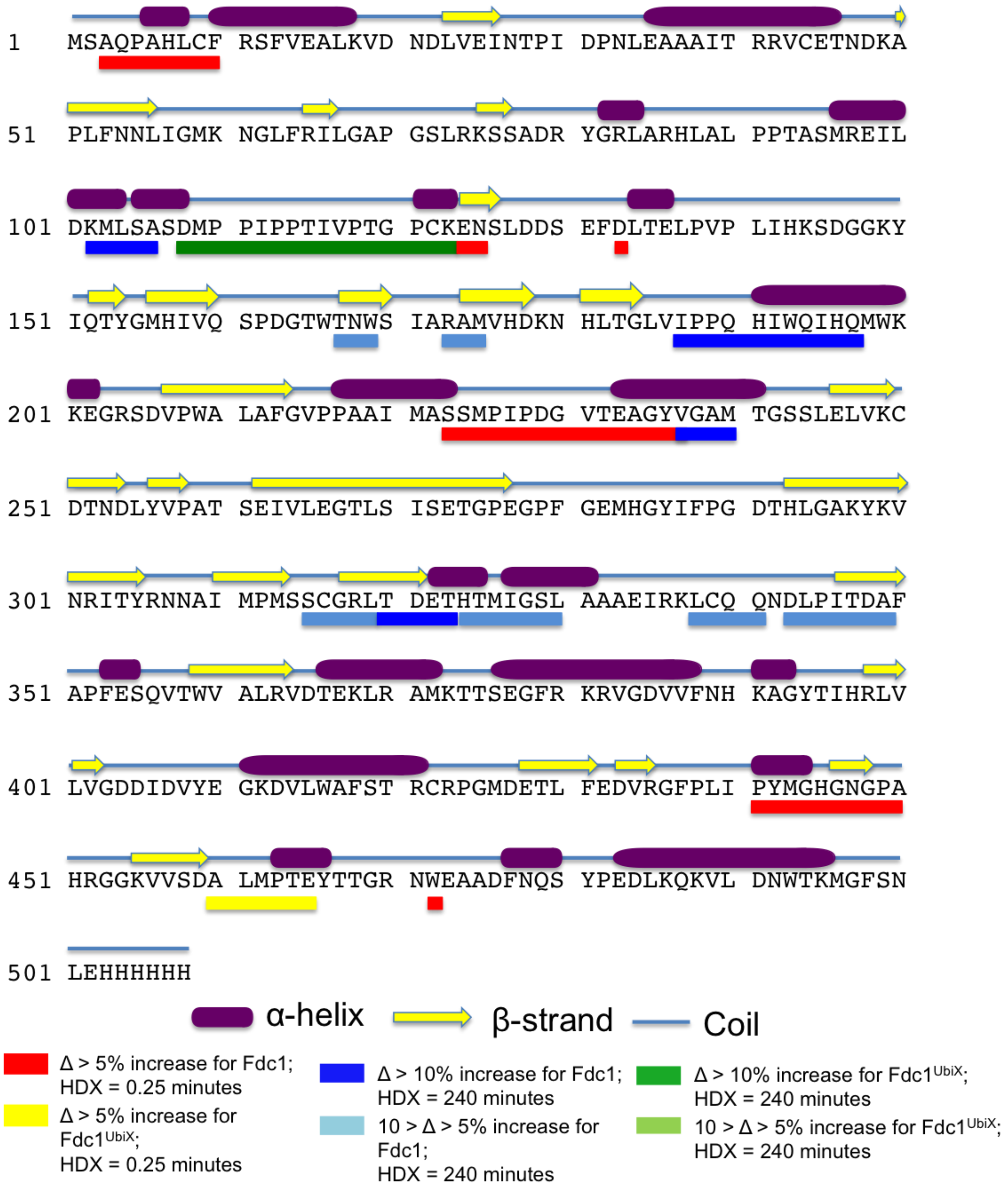

Supplementary Figure 8: Representation of structural and HDX elements on the primary sequence. The structural elements in the crystallographic model of Fdc1^{UbiX} are identified above the sequence, with line format designating α-helix/ β-strand/ random coil. The uptakes from all peptides has been combined; primarily many overlapping short peptides have been used but at times only single longer peptides are found which can be seen with reference to Supplementary Figure 5 above.

Supplementary Reference

1 Pettersen, E. F. *et al.* UCSF chimera - A visualization system for exploratory research and analysis. *J. Comput. Chem.* **25**, 1605-1612 (2004).
